# Polymorphisms in the interleukin-10 gene cluster are possibly involved in the increased risk for major depressive disorder

**DOI:** 10.1186/1471-2350-9-111

**Published:** 2008-12-16

**Authors:** Tanel Traks, Kati Koido, Triin Eller, Eduard Maron, Külli Kingo, Veiko Vasar, Eero Vasar, Sulev Kõks

**Affiliations:** 1 Department of Physiology, University of Tartu, Ravila 19, 50411 Tartu, Estonia; 2 Centre of Molecular and Clinical Medicine, University of Tartu, Tartu, Estonia; 3 Department of Psychiatry, University of Tartu, Tartu, Estonia; 4 Research Department of Mental Health, The North Estonian Regional Hospital, Psychiatry Clinic, Tallinn, Estonia; 5 Estonian Genome Foundation, University of Tartu, Tartu, Estonia; 6 Department of Dermatology and Venerology, University of Tartu, Tartu, Estonia; 7 Institute of Veterinary Medicine and Animal Sciences, Estonian University of Life Sciences, Tartu, Estonia

## Abstract

**Background:**

Innate immune inflammatory response is suggested to have a role in the pathogenesis of major depressive disorder (MDD). Interleukin (IL)-10 family cytokines IL-10, IL-19, IL-20, and IL-24 are all implicated in the inflammatory processes and polymorphisms in respective genes have been associated with various immunopathological conditions. This study was carried out to investigate whether single-nucleotide polymorphisms (SNPs) in these genes are also associated with MDD.

**Methods:**

Case-control association study was performed with seven SNPs from the *IL10 *gene cluster. 153 patients with MDD and 277 healthy control individuals were recruited.

**Results:**

None of the selected SNPs were individually associated with MDD. The linkage disequilibrium (LD) analysis indicated the existence of two recombination sites in the *IL10 *gene cluster, thus confirming the formerly established LD pattern of this genomic region. This also created two haplotype blocks, both consisting of three SNPs. Additionally, the haplotype analysis detected a significantly higher frequency of block 2 (*IL20 *and *IL24 *genes) haplotype TGC in the patients group compared to healthy control individuals (P = 0.0097).

**Conclusion:**

Our study established increased risk for MDD related to the *IL20 *and *IL24 *haplotype and suggests that cytokines may contribute to the pathogenesis of MDD. Since none of the block 2 SNPs were individually associated with MDD, it is possible that other polymorphisms linked to them contribute to the disease susceptibility. Future studies are needed to confirm the results and to find the possible functional explanation.

## Background

Major depression (MD or major depressive disorder, MDD) is a complex disease that not only affects the lives of the patients but also their family members and is one of the major causes of disability worldwide, ranking 4th according to a recent survey of global burden of disease and estimated to become 2nd by 2030 [1]. To date, the most popular explanation of the cause has been the monoamine hypothesis which only encompasses a part of the disturbances producing the disease. Hence, many new theories and prospects for pharmacotherapy are currently being developed [2]. Among these, considerable effort has been made to elucidate the role of the innate immune inflammatory response in depression with elevated pro-inflammatory cytokines influencing neurotransmitter metabolism, neuroendocrine function, synaptic plasticity and information processing [3].

Interleukin (IL)-10 and it's recently discovered paralogs IL-19, IL-20, and IL-24 are all implicated in the inflammatory processes and act on the Th1/Th2 cytokine balance [4]. All of their genes are located in the *IL10 *gene cluster in a 200 kb region of chromosome 1 within the locus q31-32. In accordance with the proposed role of these cytokines in various inflammatory diseases, the polymorphisms in respective genes have also been associated with many immunopathological conditions, especially psoriasis [5-7]. In addition, while elevated pro-inflammatory cytokines such as IL-1, IL-6 and TNF-α (tumor necrosis factor α) have been associated with MDD, IL-10 as a counteractive anti-inflammatory cytokine has been studied directly in reference to MDD. Results describing the relationship between MDD and IL-10 production have so far been inconsistent, showing increased [8,9], unchanged [10] or decreased [11] levels of IL-10 in depressed patients, and also differentiating between depressive subtypes [12]. It has been suggested that in case of the increased IL-10 it may indicate a compensatory anti-inflammatory response against generalized inflammatory state in MDD [13]. Furthermore, antidepressants have been shown to stimulate the production of IL-10 along with the reduction of the general pro-inflammatory/anti-inflammatory cytokine ratio [14]. In respect to neurotransmitter metabolism, IL-10 inhibits the nearly ubiquitously expressed indoleamine 2,3-dioxygenase (IDO), an enzyme responsible for directing tryptophan degradation along the kynurenine pathway [15], thus possibly increasing the availability of tryptophan for serotonin synthesis and decreasing the levels of neurotoxic *N*-methyl-D-aspartate (NMDA) receptor agonist quinolinic acid and NMDA antagonist kynurenic acid [16]. Finally, astrocytes which release IL-10 in the central nervous system and are especially sensitive to the apoptotic effects of quinolinic acid, have been observed to be reduced in MDD [16,17].

Considering the active role of *IL10 *cluster cytokines in inflammatory processes and the impaired immune function in MDD, and also the specific evidences linking IL-10 to MDD, the present study was aimed to investigate the possible association between genetic variations in these genes and MDD. We selected seven SNPs from the *IL10 *gene cluster to determine their individual and haplotype associations with MDD. It should also be noted that the genome-wide linkage analysis has identified the chromosome 1q31-32 region that contains the *IL10 *gene cluster as one of the susceptibility loci to bipolar disorder, known for its high comorbidity with MDD [18].

## Methods

### Study sample

Unrelated patients (n = 153; 38 males; 115 females; mean age ± SD: 40.5 ± 13.4 yr) with MDD were recruited in the study along with healthy control individuals (n = 277; 70 males; 206 females; mean age ± SD: 39.4 ± 14.0 yr) from the Estonian population. Diagnoses of patients were substantiated by psychiatric interview and verified by Mini International Neuropsychiatric Interview (M.I.N.I. 5.0.0) based on DSM-IV [19]. The case group consisted of patients with only MDD and patients with MDD and comorbid anxiety disorders (panic disorder, generalized anxiety disorder, obsessive compulsive disorder, social fobia). Controls were evaluated using M.I.N.I. to exclude those with psychiatric morbidity, and with a family history interview to exclude those with a known history of major psychiatric disorders in first-degree relatives. Patients were recruited among consecutive outpatients and in-patients at the Clinic of Psychiatry of Tartu University Hospital and controls were recruited by newspaper advertisement in Tartu, Estonia. The study was conducted in accordance with the principles of the Declaration of Helsinki. The Ethics Review Committee on Human Research of the University of Tartu approved the study protocol. Each subject provided written informed consent.

### Marker selection and genotyping

In our previous study, we identified two haplotype blocks within the *IL10 *gene cluster encompassing thirteen SNPs from *IL19*, *IL20*, and *IL24 *genes [6]. Among these, six tag SNPs (three from both blocks) were selected for this study using the Haploview Tagger program [20]. The selected SNPs of block 1 were rs2243188 and rs2243193 of *IL19 *(intron 6, 3' UTR) and rs2981572 of *IL20 *(5' near gene) and SNPs of block 2 were rs1518108 of *IL20 *(3' UTR), rs1150253 and rs1150258 of *IL24 *(intron 2, exon 5). Additionally, we included rs1800872 of *IL10 *(5' UTR) that lies within the putative STAT 3 binding site and has been associated with IL-10 expression [21,22]. All selected SNPs had a minor allele frequency above 20% and were separated by more than 1000 bp.

Genomic DNA was extracted from the whole blood and the SNPs rs2243193, rs2981572, and rs1150253 were analyzed by the tetra-primer ARMS-PCR method as described previously [6,23,24]. For the SNPs rs1800872, rs2243188, rs1518108, and rs1150258 the Applied Biosystems (Foster City, California) SNPlex™ assay was used [25]. This method is based on the oligonucleotide ligation/polymerase chain reaction assay (OLA/PCR) using allele-specific ZipCode™ probes and adaptors followed by hybridization of fluorescently labelled ZipChute™ probes that allow the detection of genotypes by capillary electrophoresis. The probes were detected with an Applied Biosystems 3730 DNA Analyzer, and data interpretation was performed with the Applied Biosystems Genemapper v4.0 software.

### Statistical analyses

Single marker association analysis and multimarker haplotype association tests between groups of patients and controls and Hardy-Weinberg equilibrium calculations in both groups were performed using the Haploview v4.0 software [20]. Analysis of genotype frequencies was performed using the web version of Genepop [26]. The Solid Spine of linkage disequilibrium (LD) method was used to define the haplotype blocks and the resulting blocks were used in the haplotype association test. The extent of disequilibrium was demonstrated by the standardized D' characteristic that was multiplied by 100 in the LD illustration generated in Haploview. One thousand permutations were used to correct P-values for multiple testing error in haplotype analysis.

## Results

Seven SNPs of the genomic region of *IL10*, *IL19*, *IL20*, and *IL24 *genes (*IL10 *cluster) were analyzed in 153 Estonian patients with MDD and 277 healthy control subjects. Genotype frequencies of these polymorphisms did not deviate significantly from the Hardy-Weinberg equilibrium in neither of the groups. Comparing genotype and allele frequencies between MDD patients and controls, none of the analyzed seven SNPs showed statistically significant association with susceptibility to MDD (Table 1).

**Table 1:** Genotype and allele frequencies of SNPs from the *IL10 *gene cluster in MDD patients (n = 153) and control individuals (n = 277)

	**Alleles**		**Genotypes**				**Alleles**		
						
**SNP ID**	1/2*	**Groups**	**11**	**12**	**22**	**P-value**	**1**	**2**	**P-value**
rs1800872	C/A	Controls	51.0	41.3	7.7	0.5460	71.6	28.4	0.5238
		Patients	53.2	41.0	5.8		73.7	26.3	
rs2243188	C/A	Controls	65.8	30.7	3.5	0.1790	81.1	18.9	0.1820
		Patients	57.1	40.0	2.9		77.1	22.9	
rs2243193	G/A	Controls	62.8	33.1	4.1	0.4803	79.3	20.7	0.4636
		Patients	59.2	35.9	4.9		77.1	22.9	
rs2981572	T/G	Controls	48.4	42.9	8.7	0.6789	69.9	30.1	0.6406
		Patients	46.4	43.6	10.0		68.2	31.8	
rs1518108	C/T	Controls	27.5	56.6	15.9	0.8755	55.8	44.2	0.8251
		Patients	29.5	51.1	19.4		55.0	45.0	
rs1150253	G/A	Controls	27.1	55.2	17.7	0.8855	54.7	45.3	0.8657
		Patients	31.9	46.8	21.3		55.3	44.7	
rs1150258	T/C	Controls	27.0	56.4	16.6	0.8741	55.2	44.8	0.8227
		Patients	29.9	48.9	21.2		54.4	45.6	

* 1 represents the major allele, 2 represents the minor allele

**Figure 1:**
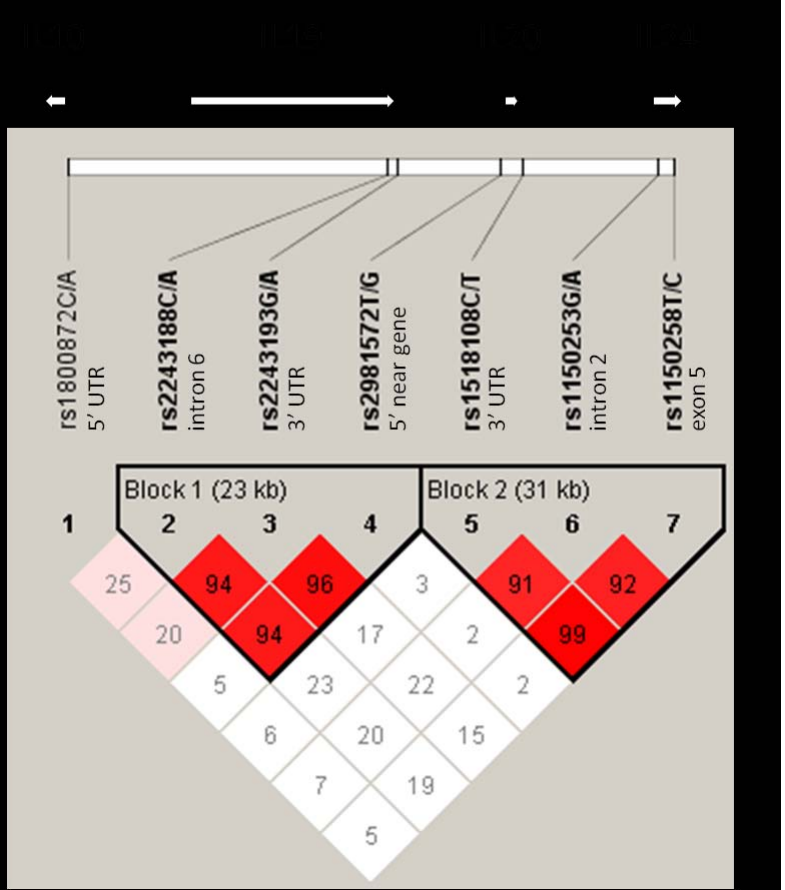
**The selected single-nucleotide polymorphisms (SNPs) of the *IL10 *gene cluster**. The relative positions and transcriptional orientation of the *IL10*, *IL19*, *IL20*, and *IL24 *genes are shown. The extent of linkage disequilibrium (LD) is demonstrated by the D' characteristic multiplied by 100 and haplotypes identified using the Solid Spine of LD method are boxed.

Haplotype analysis of the *IL10*, *IL19*, *IL20*, and *IL24 *genes was performed according to the pairwise linkage disequilibrium pattern observed within each of these genes (cases + controls, n = 429). The LD analysis indicated the existence of two recombination sites in the *IL10 *gene cluster, the first between rs1800872 of *IL10 *and rs2243188 of *IL19 *(|D'| = 0.25) and the second between rs2982572 and rs1518108 of *IL20 *(|D'| = 0.03; Figure 1). This also created two haplotype blocks, one containing rs2243188, rs2243193, and rs2981572 (the first two SNPs from *IL19 *and the third SNP from *IL20 *gene; |D'| 0.94–0.96) and the other containing rs1518108, rs1150253, and rs1150258 (the first SNP from *IL20 *and the rest from *IL24 *gene; |D'| 0.91–0.99; Figure 1). Four common haplotypes with an estimated frequency ≥ 1% were identified in both blocks, together comprising 98.6% and 99.1% of all haplotypes in block 1 and block 2, respectively. Additionally, the haplotype analysis provided one haplotype significantly associated with increased disease susceptibility. Namely, the block 2 haplotype TGC frequency was significantly higher in patients with MDD compared to the control group (P = 0.0097; OR 3.45; 95% CI 1.28–9.32; Table 2) and the result remained statistically significant after permutations (P_adj _= 0.042; Table 2). However, the overall frequency of this haplotype was only 2.1% in the pooled group of patients and controls.

**Table 2:** Haplotype analysis of SNPs from the *IL10 *gene cluster with major depressive disorder.

	**Controls** **(n = 277)**	**Patients** **(n = 153)**	**P-value nom**	**P-value adj**	**OR (95% CI)**
Block 1 haplotypes (*IL19 *and *IL20 *genes)					
CGT	68.6	67.3	0.7093	1.0000	0.94 (0.69–1.28)
AAG	17.9	21.4	0.2297	0.8840	1.25 (0.87–1.79)
CGG	10.1	8.4	0.4168	0.9890	0.81 (0.49–1.35)
CAG	2.3	1.1	0.2506	0.9180	0.49 (0.14–1.68)
					
Block 2 haplotypes (*IL20 *and *IL24 *genes)					
CGT	53.3	51.6	0.6459	1.0000	0.94 (0.70–1.25)
TAC	42.8	40.8	0.5768	1.0000	0.92 (0.69–1.23)
CAT	1.9	2.8	0.4109	0.9890	1.47 (0.58–3.74)
TGC	1.2	3.9	**0.0097**	**0.0420**	3.45 (1.28–9.32)

P-values ≤ 0.05 are in bold; P-value nom: nominal P-value; P-value adj: permutation adjusted P-value; OR: odds ratio; CI: confidence interval

## Discussion

Numerous studies have established statistical associations of the *IL10 *gene cluster polymorphisms with various inflammatory diseases [5-7] and the innate immune inflammatory response is suggested to have a role in the etiology of MDD [3]. Deriving from that, the present study was intended to determine the associations between seven selected SNPs from the aforementioned genomic region and MDD.

The results indicated that none of the SNPs were individually associated with MDD. On the part of the *IL10 *gene, this was in line with a previous report also finding no association between the *IL10 *gene and MDD, analyzing a promoter polymorphism at position -819 in that case [27]. Other studies investigating the association between cytokine gene polymorphisms and MDD have lead to diverse results. No significant associations were found between the polymorphisms from *IL1B *[28], *IL6 *[29] and *TNFB *[30] genes and MDD, although there was a trend for patients who were homozygous for the -511T allele of the *IL1B *gene to have less severity depressive symptoms and more favourable fluoxetine therapeutic response than -511C carriers [28]. However, polymorphisms from *TNFA *[31] and *CCL2 *[32] genes were associated with MDD. Also, single polymorphisms and seven haplotypes from inflammation-related genes involved in T-cell functioning *PSMB4 *and *TBX21 *were associated with MDD susceptibility [33].

The LD analysis indicated the existence of two recombination sites in the *IL10 *gene cluster, the first between rs1800872 and rs2243188 (|D'| = 0.25) and the second between rs2982572 and rs1518108 (|D'| = 0.03), thus confirming the formerly established LD pattern of this genomic region [6,34]. The resulting two haplotype blocks consisted of rs2243188, rs2243193, and rs2982572 (|D'| 0.94–0.96); and rs1518108, rs1150253, and rs1150258 (|D'| 0.91–0.99). Additionally, the haplotype analysis revealed that the frequency of the block 2 haplotype TGC was significantly higher among patients compared to controls and was therefore associated with susceptibility to MDD (P = 0.0097; OR 3.45; 95% CI 1.28–9.32). However, this result should be approached with caution, as the overall frequency of this haplotype was only 2.1% in the pooled group of patients and controls. The block 2 contained one SNP from the *IL20 *gene and two from the *IL24 *gene and since these SNPs weren't individually associated with MDD, it is possible that the polymorphisms contributing to the increased disease susceptibility are linked to them in the same haplotype block. Considering the elevated levels of inflammatory mediators in MDD and the mainly pro-inflammatory nature of IL-20 and IL-24 cytokines, these functional polymorphisms could affect the immunological states in the context of MDD. In addition, the block 2 extends further from the *IL24 *gene in 3' direction and includes the genes *FAIM3*, *PIGR*, and *FCAMR *(HapMap, genome build 35), that could also be affected by alternative haplotypes. Unfortunately, specific causal explanations connecting SNPs and MDD are beyond the scope of this research and could be established in functional studies.

## Conclusion

The present study established increased risk for MDD related to the *IL20 *and *IL24 *haplotype and suggests that cytokines may contribute to the pathogenesis of MDD. Since none of the block 2 SNPs were individually associated with MDD, it is possible that other polymorphisms linked to them contribute to the disease susceptibility. The main limitation in this case was the small sample size that hinders the detection of small effects. Confirmative study with increased number of the SNPs and a larger sample is needed.

## Competing interests

The authors declare that they have no competing interests.

## Authors' contributions

TT participated in the molecular genetic studies, performed the statistical analyses and drafted the manuscript. KK participated in the design of the study and the molecular genetic studies, performed the statistical analyses and helped to revise the draft. TE and EM coordinated the collection of the blood samples of the study participants and performed their psychiatric testing. KK participated in the design of the study and helped to revise the draft. VV, EV, and SK conceived the study, participated in its coordination and draft revision. All authors have read and approved the final manuscript.

## Pre-publication history

The pre-publication history for this paper can be accessed here:


